# Analysis of the effectiveness of arthroscopic cannulated screw fixation for coracoid process fractures

**DOI:** 10.3389/fsurg.2026.1790148

**Published:** 2026-07-16

**Authors:** Yang Yang, Fang Chen, Chao Feng, Qiyuan Pan, ZhiJun Zhao

**Affiliations:** 1 Department of Orthopedics, People’s Hospital of Yubei District, Chongqing, China; 2 Department of Infectious Disease, People’s Hospital of Yubei District, Chongqing, China

**Keywords:** acromioclavicular joint, coracoid process fracture, hollow screw, minimally invasive surgery, shoulder arthroscopy

## Abstract

**Objective:**

To evaluate the effectiveness of minimally invasive arthroscopic treatment for coracoid process fractures.

**Methods:**

This retrospective study analyzed 17 patients with coracoid process fractures treated between January 2020 and October 2025, comprising 8 patients managed with shoulder arthroscopy (Arthroscopy group) and 9 patients treated via open surgery (Open group). Preoperative baseline characteristics, including gender, age, body mass index(BMI),injury mechanism, injury type, Eyres classification, and concomitant rotator cuff injuries, showed no significant differences between groups (*P*>0.05). We recorded operative duration, intraoperative blood loss, postoperative hospitalization, screw placement accuracy, fracture healing time, and complications. Treatment outcomes were evaluated using Visual Analog Scale (VAS), Constant-Murley, and university of California at Los Angeles shoulder rating scale (UCLA) scores.

**Results:**

The arthroscopy group demonstrated significantly reduced intraoperative blood loss and shorter postoperative hospitalization compared to the open group (*P*<0.05). Both groups showed significant postoperative improvement in VAS, Constant-Murley, and UCLA scores relative to preoperative values (*P* < 0.05). The arthroscopy group achieved superior VAS scores at 3 days and 3 months postoperatively compared to the open group (*P* < 0.05). Furthermore, Constant-Murley and UCLA scores improved more substantially in the arthroscopy group at 3 and 6 months postoperatively (*P* < 0.05). However, no significant intergroup differences emerged in these scores at 12-month follow-up (*P* > 0.05). Screw placement accuracy was comparable between groups (*P* > 0.05), with excellent rates of 75% (6/8) in the arthroscopy group and 77.8% (7/9) in the open group. Operative time and fracture healing time showed no significant intergroup differences (*P* > 0.05). During the follow-up period ranging from 10 to 21 months (mean 15.5 months), no screw displacement or pull-out occurred. Arthroscopic procedures identified one crescent-shaped supraspinatus tear repaired with suture anchors, while two minor superficial supraspinatus tendon tears received debridement. Neither group experienced neurovascular injuries or infections.

**Conclusion:**

Compared to traditional open surgery, arthroscopic minimally invasive treatment of coracoid process fractures achieves comparable accuracy in screw placement, operative duration, and fracture healing time. The arthroscopic approach, however, results in less intraoperative bleeding and a shorter postoperative hospital stay. It also leads to more pronounced improvements in postoperative pain and shoulder function. Furthermore, it enables the concurrent treatment of associated shoulder pathologies, such as rotator cuff injury, acromial impingement, or glenohumeral joint lesions.

## 1 Introduction

Scapular fractures represent only 1% of all fractures (1), and coracoid fractures comprise merely 3∼13% of scapular fractures (2). Most coracoid fractures occur alongside injuries to the ipsilateral shoulder complex, making isolated coracoid fractures relatively uncommon. Associated injuries, such as acromioclavicular joint separation, rotator cuff tears, and fractures of the acromion, scapula, and distal clavicle, are frequent, with acromioclavicular joint separation being the most prevalent (3–5). These high-energy injuries often compromise the superior shoulder suspensory complex (SSSC), potentially resulting in shoulder dysfunction. Anatomical reduction of the coracoid fracture is therefore essential for preserving the integrity of the coracoacromial arch (6). Current surgical indications for coracoid fractures encompass painful nonunion, fracture displacement exceeding 1 cm, or multiple injuries involving the SSSC. Given the deep location and challenging exposure of coracoid fractures, open reduction with screw internal fixation has traditionally been the primary treatment (7). Recent advances in shoulder arthroscopy, however, have led several authors to report techniques for arthroscopic management of coracoid fractures with favorable outcomes. They highlight the benefits of arthroscopy's minimally invasive nature and its capacity to address concomitant intra-articular pathologies (8–11). Current literature on the arthroscopic treatment of coracoid fractures consists mainly of case reports, with a notable absence of comparative studies evaluating different surgical techniques. We therefore performed a retrospective clinical study comparing minimally invasive arthroscopic-assisted small-incision surgery with the open surgical approach for coracoid fractures.

### 1.1 Clinical data

#### 1.1.1 General information

Patient inclusion criteria comprised: ① imaging examinations demonstrating coracoid process fracture displacement ≥1 cm, classified as Eyres type III, IV, or V (4); ② coracoid process fracture combined with SSSC injury (7); and ③ Shoulder arthroscopy, the preferred preoperative approach for patients with rotator cuff injuries or other intra-articular pathologies, or traditional open surgery was used for hollow screw internal fixation; all patients provided informed consent prior to surgery. Exclusion criteria were: ① a history of pre-existing shoulder disease or severe dysfunction; ② poor compliance precluding participation in rehabilitation training; and ③ pathological fracture. From January 2020 to October 2025, 29 patients with coracoid process fractures were admitted to the Department of Orthopedics and Joint Surgery at Yubei District People's Hospital, of whom 17 met the criteria and were enrolled. Minimally invasive arthroscopic treatment (Arthroscopy group, *n* = 8) and open surgery (Open group, *n* = 9) were performed. Preoperative baseline characteristics, including gender, age, BMI, cause of injury, type of injury, Eyres classification, and concomitant rotator cuff injury, showed no statistically significant differences (*P* > 0.05) between the groups, indicating comparability (Table 1).

**Table 1:** Comparison of baseline data between the two groups.

Baseline data	Arthroscopic group	Open group	Statistical value	*P*
Gender (male/female)	6/2	8/1	**—**	0.608
Year ($\bar{x}$ ± *s*)	46. 27 ± 10.63	41.85 ± 11.39	*t* = 0.824	0.423
BMI ($\bar{x}$ *±* *s*,kg/m^2^)	24.19 ± 10.27	22.87 ± 11.95	*t* = 0.243	0.812
Cause of injury (fall/traffic accident/ crushing of heavy objects)	4/2/2	5/3/1	**—**	0.634
Type of injury (simple coracoid process fracture/SSSC injury)	3/5	2/7	**—**	0.632
Eyres type（ⅢA/IIIB/IVA/IVB/VA）	2/2/2/1/1	3/2/2/0/2	**—**	0.353
Rotator cuff injury (case,%)	3 (37.5)	2 (22.2)	**—**	1.000

#### 1.1.2 Surgical technique

All procedures in both groups were performed by the same lead surgeon.

The Open Reduction group included isolated coracoid process fractures (2 cases) and coracoid process fractures combined with acromioclavicular (AC) joint dislocation [7 cases, comprising 5 Rockwood type II and 2 type III dislocations (7)]. After satisfactory anesthesia, routine disinfection and draping were performed with the patient placed in the beach-chair position. A vertical incision was made 1 cm lateral to the coracoid process. The skin and subcutaneous tissue were sequentially dissected, and the deltopectoral interval was developed to reach the coracoid process. The overlying deltoid fibers were split to expose the fracture site. Under direct visualization, the fracture was reduced and a 1.0-mm Kirschner wire (K-wire) served as a guide pin. A 3.5-mm cannulated lag screw was then inserted for fixation, with screw and fracture positions confirmed under fluoroscopy. For the dislocated Rockwood type III AC joints, reduction and fixation were performed using K-wires, while Rockwood type II dislocations were not fixed.

Arthroscopic Group: This group comprised isolated coracoid process fractures (3 cases) and coracoid process fractures combined with AC joint dislocation (5 cases, all Rockwood type II). Standard posterior and anterolateral portals were established to explore the intra-articular space and address any associated pathologies. The base of the coracoid process was exposed intra-articularly following cauterization. The arthroscope was then transitioned to the subacromial space, where the subacromial bursa was debrided. Anterolateral and distal anterolateral portals were established to expose the rotator cuff interval, subscapularis tendon, and the base and superior surface of the coracoid process along with the coracoacromial ligament. The fracture site was exposed and the hematoma within the fracture gap was cleared. A suture was passed through the conjoined tendon superior to the coracoid, and traction on this suture mobilized the coracoid tip for approximation with the base. After achieving relative reduction, a long spinal needle was introduced percutaneously from the superior aspect of the coracoid near the acromion to determine the optimal screw trajectory. A small skin incision was made, and a guide pin was inserted into the coracoid. Once the pin penetrated the proximal fracture line, it served as a joystick, working in concert with traction on the pre-placed suture in the conjoined tendon to achieve anatomical reduction of the fracture fragments. The guide pin was then advanced into the distal fragment. Its position was verified under fluoroscopy, and a 3.5-mm diameter cannulated screw was inserted over the guide pin. Final screw position and AC joint reduction were confirmed fluoroscopically. Following coracoid fracture reduction, the Rockwood type II AC joint dislocations were essentially reduced and required no additional fixation.

#### 1.1.3 Postoperative rehabilitation protocol

Patients with combined Rockwood type III AC joint dislocations maintained temporary K-wire fixation for four weeks post-reduction. The K-wires were subsequently removed during an outpatient visit. Throughout this period, the affected shoulder was immobilized with a collar-and-cuff sling. Passive shoulder range of motion exercises commenced at four weeks postoperatively, followed by active exercises at six weeks. For all other patients, passive shoulder exercises began at two weeks postoperatively, active exercises at six weeks, and resisted training at twelve weeks.

#### 1.1.4 Outcome measures

**Figure 1:**
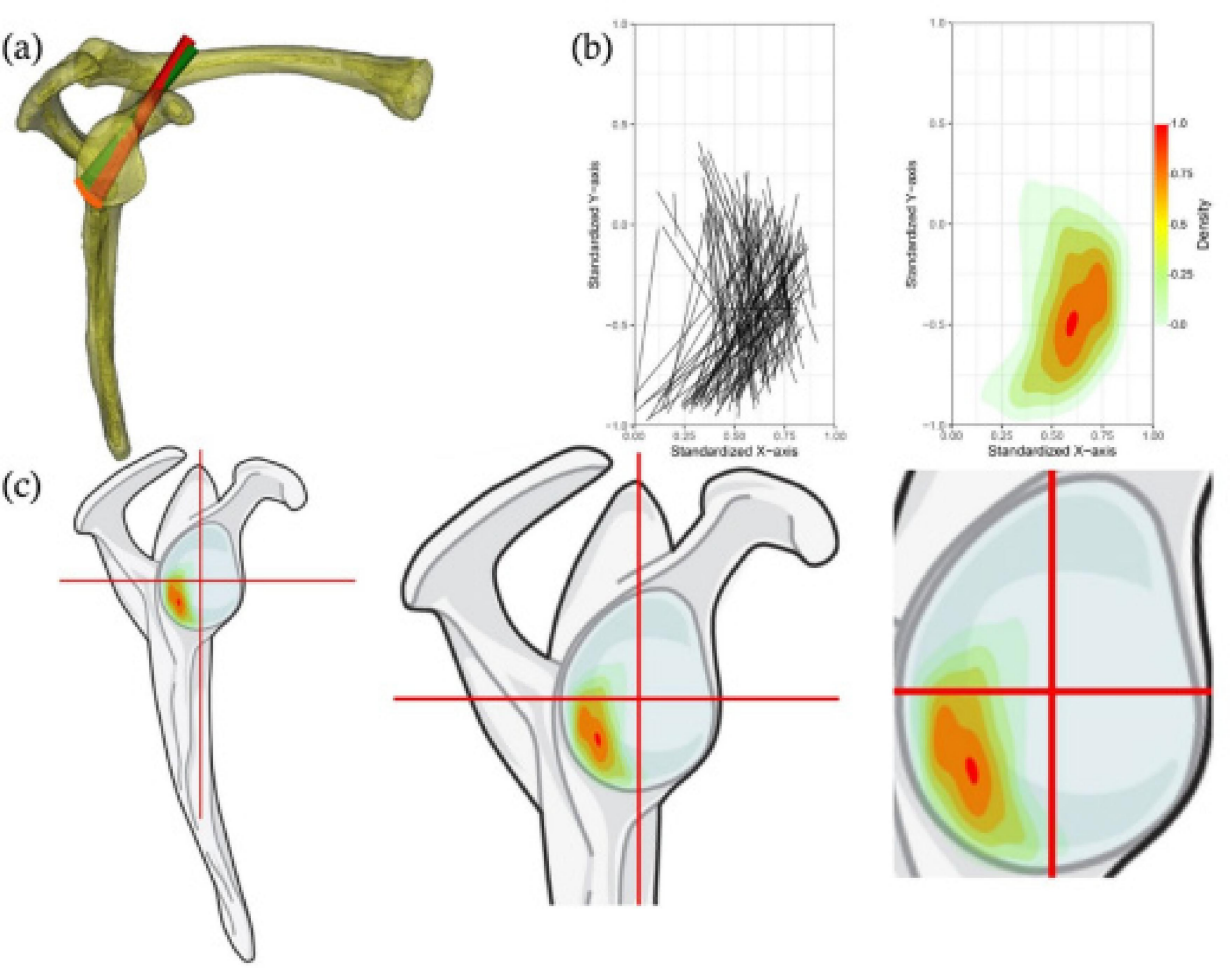
PSPA in the Y-view of the scapula. **(a)** The orange fan-like arc shows countless screw tips conforming to the screw placement rules. **(b)** After standardization based on the width of the glenoid along the X- and Y-axes, each line segment in the scatterplot represents the screw-tip positions of a patient. The scatterplot was then converted into a heatmap with a color scale indicating the relative density of screw tip positions. **(c)** The red and orange areas shown in the heatmap were defined as the PSPA, the yellow area as the CSPA, and the green and other areas as the DSPA. [Figure 1 from manuscript Wang X, Wang Y, Xu H, Yin Z, Liang B, Yan J, et al. Preferred screw placement area identified by imaging based quadrant and heatmap analysis for long screw fixation in Ogawa I coracoid process fractures. Sci Rep. 2025 Jul 18;15(1):26094].

We recorded operative time, intraoperative blood loss, and postoperative hospital stay. Postoperative three-dimensional CT reconstructions assessed screw placement accuracy, which was graded as excellent (within the PSPA zone), good (within the CSPA zone), or poor (within the DSPA zone) (Figure 1) (12). Follow-up x-rays documented the time to coracoid fracture union and any postoperative complications. Shoulder function recovery was evaluated during follow-up using the VAS, Constant-Murley, and UCLA score shoulder rating scale. The VAS ranges from 0 to 10, where 0-1 signifies no pain, 2-3 mild pain, 4–6 moderate pain, and 7–10 severe pain. The Constant-Murley score spans 0 to 100, and the UCLA score 0 to 35, higher scores on both scales indicate superior shoulder function.

#### 1.1.5 Statistical analysis

Statistical analyses were conducted using SPSS software (version 26.0). The Shapiro–Wilk test assessed the normality of continuous data. Normally distributed data are expressed as mean ± standard deviation ($\bar{x}$ ± s`). Independent samples t-tests were used for intergroup comparisons, while analysis of variance was employed for intragroup comparisons. Categorical data were compared using Fisher's exact test. A two-sided *α* level of 0.05 was adopted, with *P* < 0.05 considered statistically significant.

## 2 Results

All surgeries in both groups were completed successfully, with no instances of vascular or nerve injury, infection, or other complications. The Arthroscopic group exhibited significantly less intraoperative blood loss and a shorter postoperative hospital stay than the Open Reduction group (*P* < 0.05). Operative time and fracture healing time did not differ significantly between the groups (*P* > 0.05). All patients were followed for 10 to 21 months, with a mean follow-up of 15.5 months; the follow-up duration showed no significant intergroup difference (*P* > 0.05). Postoperative VAS, Constant-Murley, and UCLA scores improved significantly from preoperative values in both groups (*P* < 0.05). The Arthroscopic group achieved significantly better VAS scores at 3 days and 3 months postoperatively compared to the Open Reduction group (*P* < 0.05). Furthermore, the Arthroscopic group demonstrated significantly greater improvement in Constant-Murley and UCLA scores at 3 and 6 months postoperatively (*P* < 0.05). However, no significant differences in VAS, Constant-Murley, or UCLA scores were found between the groups at 12 months postoperatively (*P* > 0.05). Postoperative imaging review showed an excellent screw placement accuracy rate of 75% (6/8) in the Arthroscopic group and 77.8% (7/9) in the Open group, the intergroup difference in the excellent/good screw placement rate was not statistically significant (*P* > 0.05). No implant displacement or pullout occurred in either group during follow-up (Tables 2, 3). In the Arthroscopic group, one intraoperative crescent-shaped supraspinatus tear was identified and repaired with suture anchors, while two other cases involved minor superficial supraspinatus tendon tears that were treated with debridement and freshening.

**Table 2:** Comparison of outcome indicators between the two groups.

Outcome indicator	Arthroscopic group	Open group	Statistical value	*P*
Operation time ($\bar{x}$ ± *s*,min)	125.38 ± 59.21	87.59 ± 46.81	*t* = 1.469	0.163
Intraoperative blood loss ($\bar{x}$ ± *s*,mL)	85.63 ± 39.94	261.63 ± 101.39	*t* = 4.590	0.0004
Postoperative hospitalization time ($\bar{x}$ ± *s*,day)	3.77 ± 1.06	7.95 ± 3.61	*t* = 3.146	0.007
Accuracy of screw implantation (excellent/good, case)	6/2	7/1	**—**	0.628
Follow-up time ($\bar{x}$ ± *s*,month)	13.97 ± 5.63	14.58 ± 8.73	*t* = 0.167	0.868
Fracture healing time ($\bar{x}$± *s*,month)	4.85 ± 2.71	5.19 ± 3.18	*t* = 0.236	0.817

**Table 3:** Comparison of postoperative functional scores.

Group			VAS			*F*	*P*		Constant			*F*	*P*		UCLA			*F*	*P*
	pre-op	post-op 3d	post-op 3m	post-op 6m	post-op12m			pre-op	post-op 3m	post-op 6m	post-op 12m			pre-op	post-op3m	post-op6m	post-op 12m		
Arthroscopic group	8.23 ± 3.23	3.38 ± 1.89	2.33 ± 0.87	1.12 ± 0.92	0.64 ± 0.29	16.11	＜0.01	18.36 ± 9.51	56.29 ± 9.47	79.53 ± 11.28	92.83 ± 7.65	74.45	＜0.01	7.85 ± 5.38	28.75 ± 4.63	31.84 ± 6.25	33.51 ± 2.63	45.70	＜0.01
Open group	8.52 ± 3.89	5.56 ± 1.09	3.26 ± 0.63	1.35 ± 0.86	0.85 ± 0.33	11.23	＜0.01	18.61 ± 8.95	40.63 ± 8.59	61.03 ± 14.37	88.52 ± 8.74	33.72	＜0.01	7.94 ± 6.27	21.62 ± 5.38	25.78 ± 4.62	32.84 ± 3.16	26.25	＜0.01
*t*	0.17	2.96	2.55	0.53	1.39			0.06	3.58	2.92	1.08			0.03	2.91	2.29	0.47		
*P*	0.87	0.01	0.02	0.60	0.18			0.96	0.002	0.01	0.30			0.98	0.01	0.03	0.64		

pre-op, preoperative; post-op, postoperative; d, day; m, mont.

## 3 Discussion

### 3.1 Analysis of the efficacy of arthroscopic minimally invasive treatment for coracoid process fractures

Passaplan et al. first employed shoulder arthroscopy to treat Ogawa type I coracoid process fractures and detailed arthroscopic fracture reduction techniques (9). Subsequent case reports by Bishai and Xie et al. further supported the favorable outcomes of this arthroscopic approach (10, 11). To determine whether shoulder arthroscopy provides advantages over traditional open reduction in operative duration, surgical trauma, and postoperative recovery, this study compared intraoperative blood loss, operative time, postoperative hospital stay, and pain and functional scores. The arthroscopy group exhibited significantly less intraoperative blood loss and a shorter postoperative hospital stay than the open reduction group, indicating superior minimally invasive characteristics and enhanced early postoperative recovery. Operative time, however, did not differ significantly between groups, possibly because the arthroscopic procedure included additional time to address concomitant intra-articular and extra-articular pathologies. Postoperative functional assessments at 3 and 6 months revealed better scores in the arthroscopy group, which also reported significantly lower pain levels at 3 days and 3 months post-surgery. These findings underscore the role of arthroscopy in facilitating early rehabilitation and a quicker return to daily activities. By the final follow-up, however, functional outcomes were comparable between the two groups. The abundant muscular blood supply around the coracoid process and its robust self-repair capacity likely contribute to these similar long-term results, which also explains why conservative management often yields satisfactory long-term outcomes for such fractures (6). In one arthroscopy case, a crescent-shaped supraspinatus tear was identified and repaired concurrently using the suture-bridge technique, obviating the need for a second operation. This case illustrates the broader benefits of shoulder arthroscopy, including reduced tissue trauma, diminished blood loss, and accelerated recovery, alongside its capacity to manage concomitant injuries like rotator cuff tears and subacromial pathology.

**Figure 2:**
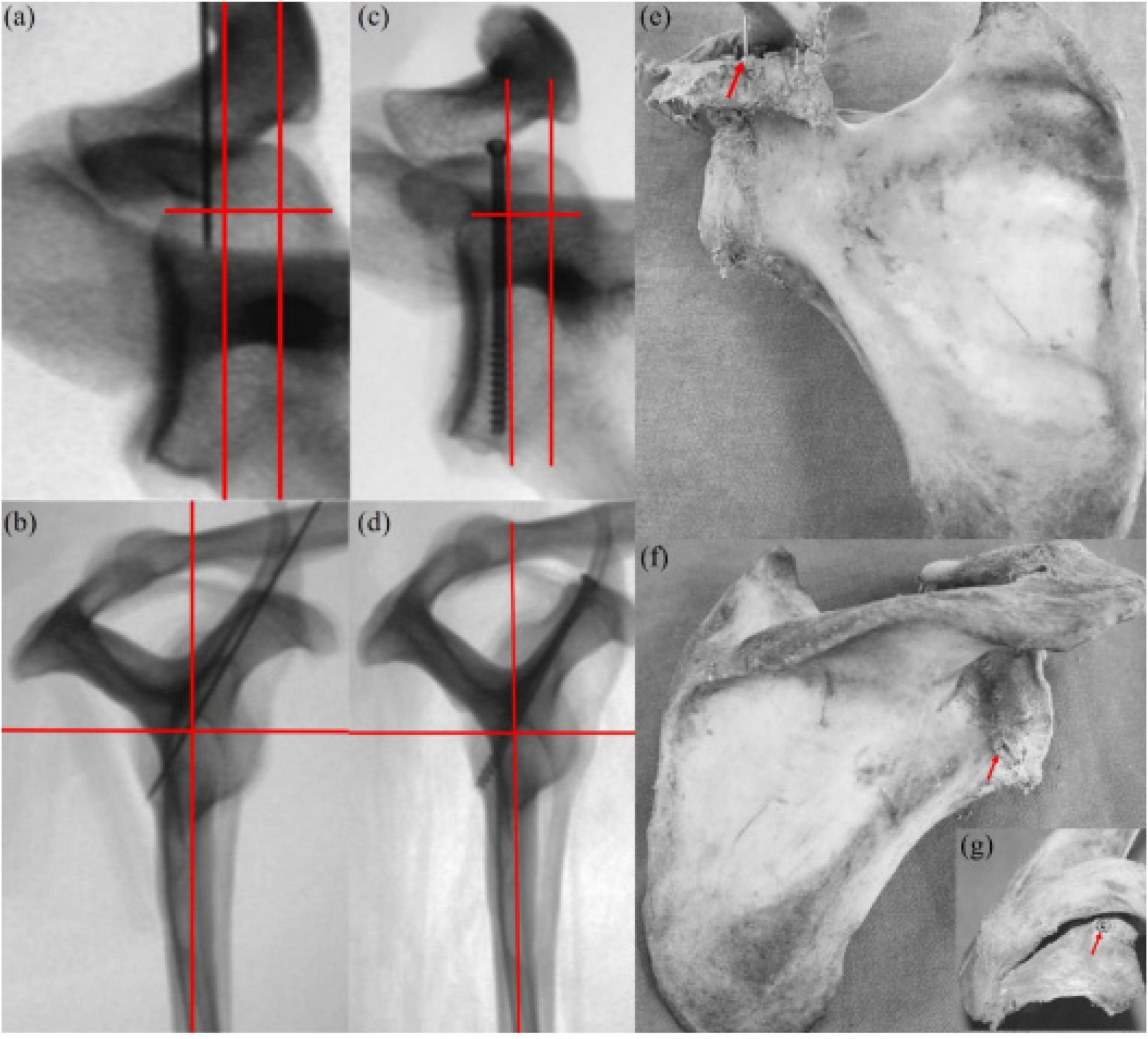
Veriffcation of the cadaveric bone of the scapula and clavicle integration model for screw placement. **(a)** Anteroposterior ffuoroscopy image of the scapula with the guide wire placed parallel to the glenoid fossa (within the lateral 1/3 of the coracoid base). **(b)** Y-view ffuoroscopy image of the scapula with the guide wire in the inferior posterior quadrant. **(c)** Anteroposterior ffuoroscopy image of the scapula with the screw placed parallel to the glenoid fossa (within the lateral 1/3 of the coracoid base). **(d)** Y-view ffuoroscopy image of the scapula with the screw in the inferior posterior quadrant. **(e)** Exterior photo of the guide wire in the anterior position (the red arrow shows the guide wire). **(f)** Exterior photo of the posterior exit point of the screw (the red arrow shows the screw tip). **(g)** Exterior photo of the entry point (the red arrow shows the screw). [Figure 2 from manuscript Wang X, Wang Y ,Xu H, Yin Z, Liang B, Yan J. Preferred screw placement area identified by imaging based quadrant and heatmap analysis for long screw fixation in Ogawa I coracoid process fractures. Sci Rep. 2025 Jul 18;15(1):26094.].

**Figure 3:**
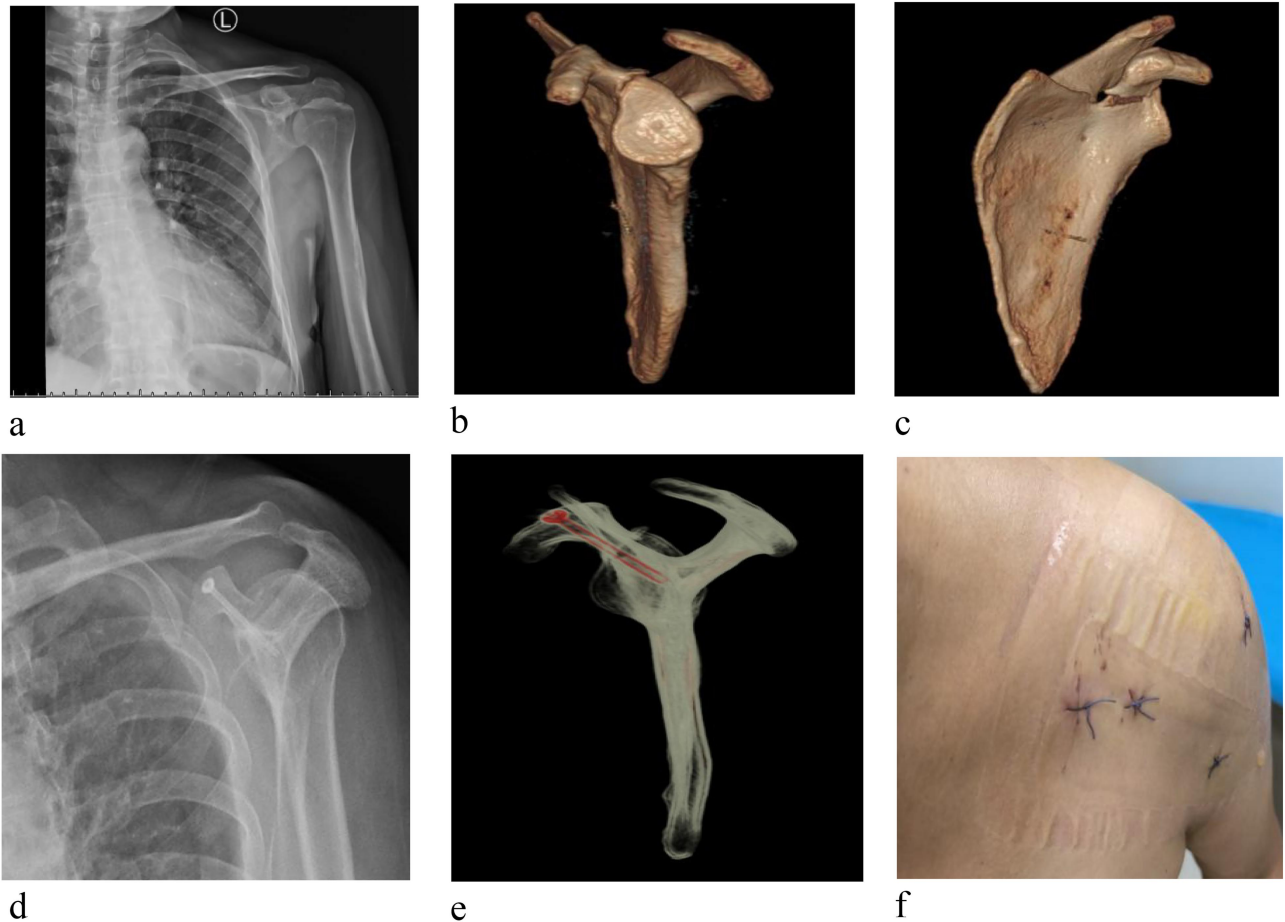
A 51-year-old female was admitted with left shoulder pain following a fall. **(a)** Preoperative image showing acromioclavicular joint dislocation (Rockwood type II). **(b,c)** Coracoid process fracture (Ogawa type I, Eyres type IV A). **(d)** Postoperative Y-view radiograph of the shoulder demonstrating reduction of the acromioclavicular joint. **(e)** Adequate fixation of the coracoid process fracture with one 3.5 mm screw. **(f)** Postoperative photograph of the surgical incision.

Through three-dimensional modeling analysis, Wang et al. identified an optimal screw entry point for coracoid process fractures, situated 2 mm anterior to the anterior edge of the conoid portion of the coracoclavicular ligament and at the lateral edge of the coracoid's superior surface near the acromial end (Figures 2, 3). This location is readily palpable during surgery and lies posterior to the conoid ligament's attachment, potentially minimizing ligament injury risk. Positioned within the clavicular concavity, it also reduces obstruction and permits more flexible screw trajectory adjustments. The recommended fluoroscopic standard entails a screw direction parallel to the glenoid tangent on the scapular anteroposterior view, with the guide pin positioned within the lateral third of the coracoid base (Figure 2). Adhering to these techniques improves the consistency of screw placement among surgeons, thereby enhancing surgical safety and fixation reliability. Their analysis determined that screw lengths for coracoid process fractures range from 40 to 53 mm, with males generally requiring longer screws than females. The study also defined a preferred screw placement area (PSPA), where screws achieve high holding power with low neurovascular injury risk (12). Accordingly, this study applied Wang et al.'s method to assess the excellent/good screw placement rate. Results indicated that one screw in the arthroscopy group and two in the open reduction group were located in the CSPA, while all others were within the PSPA. No screws were placed in the DSPA. The excellent/good screw placement rate did not differ significantly between groups, and no screw pull-out occurred. The superior visualization of the fracture site under arthroscopy likely facilitated the placement of cannulated screws as perpendicular as possible to the fracture line under direct vision. Given the coracoid process's deep location and associated fluoroscopic challenges, achieving accurate screw placement is difficult. Traditional open reduction requires not only proficient fluoroscopy but also often depends on the surgeon's tactile feedback to assess reduction, potentially leading to eccentric screw fixation, inadequate reduction, or iatrogenic neurovascular injury (8). Since the narrowest segment of the coracoid base measures only 6.6 mm (13), placing two screws demands greater technical precision, as multiple attempts can enlarge the screw pathway and compromise holding power (14). Wang et al. further suggested that double-screw fixation increases technical difficulty due to clavicular obstruction, prolongs operative time, and elevates neurovascular injury risk (12). Consequently, all cases in this study were fixed with a single 3.5 mm cannulated screw, which yielded satisfactory fracture healing and fixation strength in all patients. We therefore concur with Wang et al. that double-screw fixation should be reserved for patients with a preoperatively measured large coracoid base or those undergoing intraoperative robotic-assisted surgery (12).

Isolated coracoid process fractures are relatively rare and frequently occur with injuries to the SSSC. For patients with concomitant AC joint dislocation, Passaplan et al. reported that if the coracoclavicular ligament remains intact, reducing the coracoid process fracture alone can improve the condition without additional AC joint stabilization (9). Among the 10 patients in this study with concomitant Rockwood type II AC joint dislocations, fluoroscopy after coracoid reduction and fixation showed essentially reduced AC joints. Preoperative imaging confirmed coracoclavicular ligament continuity; thus, no internal fixation was applied to the AC joint during surgery. Two patients with Rockwood type III AC joint dislocations received supplemental Kirschner wire fixation of the AC joint following coracoid reduction. No recurrent AC joint dislocations were observed during follow-up. We therefore suggest that for Rockwood type II AC joint dislocations with intact coracoclavicular ligaments, satisfactory joint alignment after coracoid fracture reduction may render additional fixation unnecessary. This approach reduces surgical steps and operative time while avoiding potential long-term AC joint mobility limitations and the risks associated with a more extensive procedure.

### 3.2 Considerations for arthroscopic treatment of coracoid process fractures

① The distance between the coracoid process and adjacent neurovascular structures varies with patient positioning and arm position, with the beach-chair position providing the greatest separation (15). This position also better accommodates the substantial fluoroscopic demands of coracoid fracture fixation, thereby improving screw placement accuracy. We therefore recommend the beach-chair position for arthroscopic fixation of coracoid process fractures, although the final choice may depend on the surgeon's training and preference. ② Intraoperatively, the coracoid base must be fully exposed while protecting the adjacent brachial plexus, axillary artery, and axillary vein. The fracture line should be clearly visualized. A combination of anterolateral and posterior portals facilitates observation of fracture reduction and screw placement. Reduction can be assisted using Polydioxanone Suture for traction around the conjoined tendon or with a guide pin. After placing the guide pin and screw, fluoroscopy should confirm that the screw lies within the PSPA as much as possible, avoiding penetration into the joint cavity or slippage outside the scapula. If uncertain, multiple fluoroscopic angles are recommended to verify screw position within the PSPA. ③ Preoperative three-dimensional CT reconstruction is recommended to measure the coracoid base size, which helps avoid multiple intraoperative screw placement attempts that could loosen the screw pathway and compromise holding power.

## 4 Conclusion

Compared to traditional open surgery, arthroscopic minimally invasive treatment of coracoid process fractures achieves comparable accuracy in screw placement, operative time, and fracture healing duration, while offering reduced intraoperative bleeding, shorter postoperative hospitalization, and more pronounced early pain relief and functional improvement. This approach avoids the substantial soft tissue dissection and exposure challenges inherent in open procedures, which can increase trauma and complicate reduction. Arthroscopy also enables the concurrent management of associated shoulder pathologies, such as rotator cuff injuries, acromial impingement, or glenohumeral lesions, thereby significantly shortening the overall rehabilitation period and eliminating the need for subsequent operations. The limitations of this study include its single-center retrospective design, the relatively small number of cases, and the absence of biomechanical validation. Future work should involve prospective, multi-center trials with larger sample sizes and incorporate biomechanical testing to further substantiate these findings.

## Data Availability

The raw data supporting the conclusions of this article will be made available by the authors, without undue reservation.
